# 5^th^ International AIDS Society Conference on HIV Pathogenesis, Treatment and Prevention: summary of key research and implications for policy and practice – Basic sciences

**DOI:** 10.1186/1758-2652-13-S1-S2

**Published:** 2010-06-01

**Authors:** Mark Mascolinli, Rodney Kort

**Affiliations:** 1 Allentown, 18102, USA; 2 Kort Consulting, Toronto, M4Y 2T6, Canada

## Abstract

Basic science studies at the 5^th^ IAS Conference on HIV Pathogenesis, Treatment and Prevention (IAS 2009) provided important new information that has implications not only for treatment, but also for better understanding the complex dynamics of HIV infection, epidemiology, and the impact of biology and genetics on vulnerability to HIV infection, disease progression and the risk of vertical transmission. There was renewed interest in strategies on how to eliminate residual viremia, bolster the immune system and potentially achieve viral eradication given recent evidence that antiretroviral therapy (ART) is effective at minimizing viral reservoirs if administered early in acute infection.

## Discussion

Summarizing Track A reports, lead rapporteur Wendy Burgers (University of Cape Town) highlighted invited lectures on viral reservoirs and eradication, immune activation, acute infection and cellular immunity [1]. In the first of those four fields, Jean-Pierre Routy (McGill University, Montreal) analyzed strategies to mobilize reservoirs that contain virus beyond the reach of standard ART [2], including studies of valproic acid, histone deacetylase inhibitors and NF-kappa-B-independent activators. He also reviewed work on interleukin-7 as an agent to prevent viral latency and promote immune reconstitution.

Routy concluded that early ART “represents the easiest intervention to control reservoir size”, a concept explored further by Joep Lange (University of Amsterdam) [3]. Lange noted that a seminal study of viral decay rates estimated it would take 7.7 years of suppressive ART to eliminate HIV from resting CD4+ T cells in blood, although HIV persistence at other sites, such as the gut, may continually seed new reservoirs [4]. But Lange maintained that treatment soon after infection could make those reservoirs smaller. In an observational study, all nine patients who began ART before HIV seroconversion and six of eight who began within six months of seroconversion had no detectable virus in cell reservoirs, compared with all 17 comparison patients who began ART during chronic HIV infection [5]. 

In a scientific keynote address, Nobel Laureate Françoise Barré-Sinoussi (Pasteur Institute, Paris) addressed the issue of viral persistence in cellular reservoirs [6]. She proposed that ART may need to be started earlier in the course of infection and followed with a discussion of strategies that restore the immune system (to prevent immune senescence) and that target residual infected cells (to limit residual disease). Barré-Sinoussi, an IAS Governing Council member, will chair a two-day basic science workshop on controlling HIV reservoirs before the next International AIDS Conference, scheduled for July 2010 in Vienna.

IAS 2009 also featured several compelling studies on genetic and cellular research affording new insights on HIV infection risk in African women, viral loads in HIV-1 subtype C-infected people, breast milk transmission of HIV, and overall vertical transmission risk. 

### Biological factors behind HIV susceptibility and vertical transmission

Comparing women in Kisumu, Kenya, and San Francisco, USA, Craig Cohen (University of California, San Francisco) documented higher proportions and numbers of activated CD4 cells, the primary target of HIV, in the genital tract of women in Kisumu [7]. Until this study, the higher HIV risk in African women than in women elsewhere and in African men has been attributed primarily to socio-behavioural and gender norms and to high rates of sexually transmitted infections (STIs).

This study of 18- to 24-year-old women without HIV or other STIs found significantly higher levels and/or proportions of seven activated T-lymphocyte subtypes, including activated CD4 and CD8 cells. Compared with the 18 women in San Francisco, the 36 in Kisumu also had significantly higher concentrations of a cytokine that favours HIV transmission and significantly lower concentrations of two immune factors that protect from HIV. The investigators speculated that higher levels of activated T-lymphocytes in African women may reflect their greater exposure to pathogens, including parasites and viruses.

**Figure 1:**
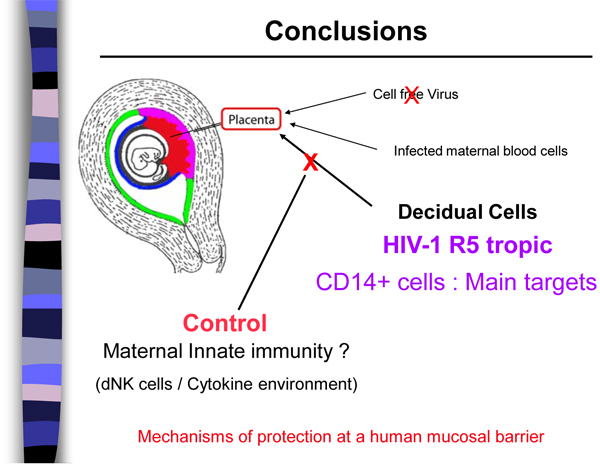
**Mechanisms of protection at a human mucosal barrier** Source: Marlin R, et al: **Antigen-presenting cells represent potential targets for R5 tropic HIV-1 infection in the first trimester pregnancy human uterine mucosa.** 5^th^ IAS Conference on HIV Pathogenesis, Treatment and Prevention: Cape Town, South Africa. MOAA204 [8]

Innovative research by Romain Marlin (Pasteur Institute, Paris) showed that cells of the maternal uterine mucosa efficiently transfer HIV-1 to other cells, such as placental cells (Figure 1) [8]. Yet HIV-1 transmission remains rare in utero, especially during the first trimester. To evaluate viral susceptibility and transmission in the uterine mucosa, Marlin exposed mucosal cells to HIV-1 that uses the CCR5 coreceptor or the CXCR4 coreceptor. The tissue tested came from HIV-negative women who had elective abortions. CD14-expressing cells proved the main target of CCR5-using HIV-1, the type of virus usually involved in HIV-1 transmission. Although infected CD14 cells produce low levels of virus, they efficiently transfer virus to other cells. What stifles viral transmission from the uterine mucosa to placenta remains a mystery (Figure 1). Discovering the factor or factors involved in preventing that transmission could offer clues important to other areas of prevention research.

### Genetic and cellular variables affecting HIV progression and vertical transmission

In a study of 427 ART-naive Zulu and Xhosa people infected with HIV-1 subtype C, the most prevalent subtype in the world, Boris Julg (Ragon Institute, Boston, and University of KwaZulu-Natal) found that the 16 individuals (4%) expressing the HLA DRB1*1303 allele had significantly lower viral loads than people without that gene (about 8500 versus 43,000 HIV-1 RNA copies/mL) [9]. The correlation held true after statistical adjustment for expression of HLA B57. (HLA class II molecules are involved in the presentation of antigens to T cells.) The protective activity of the *1303 allele did not correlate with increased T cell functional responses, a finding suggesting that this allele may promote lower viral loads by some alternative mechanism.

A study of lactating women found that CD4 cells latently infected with HIV-1 and isolated in breast milk can be spontaneously activated, even if ART makes HIV-1 undetectable in blood [10]. Diane Valea (Centre Muraz, Bobo Dioulasso, Burkina Faso) determined that all blood and breast milk samples from six women taking ART and nine women taking a brief perinatal antiretroviral regimen contained highly activated CD4+ T cells that spontaneously secreted HIV-1 antigens and viral RNA, regardless of whether HIV-1 RNA could be detected in breast milk or blood. The findings suggest an unsuspected HIV-1 cellular reservoir that could play a pivotal role in viral transmission to breastfed infants.

In addition to maternal variables that affect risk of vertical transmission, genetic factors in infants may heighten their risk of infection, according to a study of 131 Kenyan infants by Robert Choi (University of Washington, Seattle) [11]. Ninety-two infants (75%) had the wild-type allele (C/C) at amino acid position 868 of CD4; 37 infants (19%) had the heterozygous allele (C/T); and two infants (5%) had the homozygous allele (T/T). Thirty infants (23%) became infected, seven of them after one month of age. Heterozygous infants had a two times higher overall risk of HIV infection than wild-type infants, and homozygous infants had a four times higher overall risk. Heterozygosity or homozygosity raised the risk of infection after one month (implicating breast milk transmission) almost six times.

## Conclusions

The highly productive and clinically relevant basic research presented in Track A underlines the need for greater funding and wider deployment of basic benchwork initiatives in countries with high HIV prevalence. The finding that women in sub-Saharan Africa have more activated CD4 T cells in genital tract mucosa than US women [7] demonstrates that: (1) basic research can uncover unsuspected HIV risk correlations; and (2) behavioural and cultural factors may not explain all, or even most, of the heightened HIV risk in certain populations.

The study of uterine mucosal cell susceptibility to HIV-1 illustrates the gaps that remain in understanding vertical transmission of the virus [8]. Uncovering the mechanism that limits vertical transmission in utero, despite apparently efficient cell-to-cell transfer of the virus, could have implications for prevention research beyond vertical transmission.

The finding that an HLA class II genetic factor correlates with lower HIV-1 loads in subtype C-infected people [9] shows that much remains to be learned about how genetic variables in diverse populations affect HIV-1 disease progression. The discovery that HLA DRB1*1303 exerts its influence on viral load independently of T cell functional response emphasizes that wider research in highly affected populations can expand the understanding of innate HIV-specific immunity.

The breast milk study [10] may explain why even suppressive ART does not completely stop HIV-1 transmission via breast milk, although Track B studies reviewed in this supplement [11] suggest effective therapy can cut breast milk transmission to less than 1%. Work should continue to search for alternative vertical transmission prevention strategies in antiretroviral-treated women with or without detectable HIV-1 RNA in blood. Results of the CD4 gene study [12] underline the importance of learning more about genetic cofactors that influence HIV-1 acquisition and progression as potential means of improving prevention and treatment strategies.

Finally, Track A lead rapporteur Wendy Burgers [1] suggested that reported findings that early ART can dramatically decrease the size of the latent HIV reservoir underscore the call for universal treatment from early stages of infection. Numerous studies in Track B add to the already weighty evidence supporting use of ART earlier in the course of HIV infection, particularly in countries that now use a CD4 threshold of 200 cells/mm^3^.

## Competing interests

MM and RK are independent consultants contracted by the International AIDS Society for the purpose of drafting the IAS 2009 Impact Report: Summary of Key Research and Implications for Policy and Practice.

## Authors’ contributions

MM drafted the initial text. RK adapted the text for publication in a peer-reviewed journal. Both authors have approved the manuscript for publication.

## References

[B1] Burgers W Track A rapporteur report. 5th IAS Conference on HIV Pathogenesis Treatment and Prevention 2009 WESS101

[B2] Routy JP Treatments to mobilize the reservoirs. 5th IAS Conference on HIV Pathogenesis Treatment and Prevention TUBS103

[B3] Lange J Is eradication a realistic aim? 5th IAS Conference on HIV Pathogenesis Treatment and Prevention TUBS104

[B4] Chun TW Justement JS Moir S Hallahan CW Maenza J Mullins JI Collier AC Corey L Fauci AS Decay of the HIV reservoir in patients receiving antiretroviral therapy for extended periods: implications for eradication of virus. J Infect Dis 2007 13 1762 4 PubMed Abstract 10.1086/518250 17492591

[B5] Strain MC Little SJ Daar ES Havlir DV Gunthard HF Lam RY Daly OA Nguyen J Ignacio CC Spina CA Richman DD Wong JK Effect of treatment, during primary infection, on establishment and clearance of cellular reservoirs of HIV-1. J Infect Dis 2005 13 1410 8 PubMed Abstract 10.1086/428777 15809898

[B6] Barré-Sinoussi F Can the establishment and persistence of HIV reservoirs ever be controlled? 5th IAS Conference on HIV Pathogenesis Treatment and Prevention SUSS105

[B7] Cohen C Moscicki B Scott M Ma Y Shiboski S Bukusi E Daud I Rebbapragada A Brown J Kaul R Immune activation in the genital tract may account for increased HIV susceptibility among healthy young women in sub-Saharan Africa5th IAS Conference on HIV Pathogenesis Treatment and Prevention WELBA102

[B8] Marlin R Nugeyre MT de Truchis C Berkane N Gervaise A Barré-Sinoussi F Menu E Antigen-presenting cells represent potential targets for R5 tropic HIV-1 infection in the first trimester pregnancy human uterine mucosa. 5th IAS Conference on HIV Pathogenesis Treatment and Prevention MOAA204

[B9] Julg B Moodley E Nair K van der Stok M Bishop K Reddy S Mncube Z Qi Y P Goulder P Walker B Carrington M Ndung'u T The HLA class II allele DRB1*1303 is associated with reduced viral loads, independently of HLA-B57, in an HIV-1 clade C infected African population5th IAS Conference on HIV Pathogenesis Treatment and Prevention MOPDA10

[B10] Valea DC Tuaillon E Al Taaba Y Rouet F Rubbo PA Meda N Foulongne V Bollore K Vendrell JP Van de Perre P Highly activated CD4+ T cells spontaneously producing human immunodeficiency virus type I in breast milk from women treated with antiretroviral drugs. 5th IAS Conference on HIV Pathogenesis Treatment and Prevention TUAA105

[B11] Mascolini M Kort R 5th International AIDS Society Conference on HIV Pathogenesis, Treatment and Prevention: Summary of Key Research and Implications for Policy and Practice - Clinical sciences Journal of the International AIDS Society 2010 13 Suppl 1 S3 10.1186/1758-2652-13-S1-S3 PMC2880254 20519024

[B12] Choi R Farquhar C Juno J Mbori-Ngacha D Lohman-Payne B Vouriot F Wayne S John-Stewart G Bosire R Fowke K Infant CD4 C868T polymorphism is associated with increased HIV-1 acquisition overall and via breastmilk. 5th IAS Conference on HIV Pathogenesis Treatment and Prevention TUPDA105 10.1111/j.1365-2249.2010.04096.x PMC2883118 20132229

